# The Genetic Relatedness and Antimicrobial Resistance Patterns of Mastitis-Causing *Staphylococcus aureus* Strains Isolated from New Zealand Dairy Cattle

**DOI:** 10.3390/vetsci8110287

**Published:** 2021-11-22

**Authors:** Sabrina S. Greening, Ji Zhang, Anne C. Midwinter, David A. Wilkinson, Scott McDougall, M. Carolyn Gates, Nigel P. French

**Affiliations:** 1 School of Veterinary Science, Massey University, Palmerston North 4410, New Zealand; S.McDougall@massey.ac.nz (S.M.); C.Gates@massey.ac.nz (M.C.G.); 2 Infectious Disease Research Centre, Massey University, Palmerston North 4410, New Zealand; jizhang.palmy@gmail.com (J.Z.); A.C.Midwinter@massey.ac.nz (A.C.M.); dwilkin799@gmail.com (D.A.W.); N.P.French@massey.ac.nz (N.P.F.); 3 New Zealand Food Safety Science and Research Centre, Hopkirk Research Institute, Massey University, Palmerston North 4410, New Zealand; 4 Cognosco, AnexaFVC, P.O. Box 21, Morrinsville 3330, New Zealand

**Keywords:** *Staphylococcus aureus*, mastitis, whole-genome sequencing, antimicrobial resistance, cattle

## Abstract

*Staphylococcus aureus* is one of the leading causes of bovine mastitis worldwide and is a common indication for use of antimicrobials on dairy farms. This study aims to investigate the association between on-farm antimicrobial usage and the antimicrobial resistance (AMR) profiles of mastitis-causing *S. aureus*. Whole-genome sequencing was performed on 57 *S. aureus* isolates derived from cows with either clinical or subclinical mastitis from 17 dairy herds in New Zealand. The genetic relatedness between isolates was examined using the core single nucleotide polymorphism alignment whilst AMR and virulence genes were identified *in-silico*. The association between gene presence-absence and sequence type (ST), antimicrobial susceptibility and dry cow therapy treatment was investigated using Scoary. Altogether, eight STs were identified with 61.4% (35/57) belonging to ST-1. Furthermore, 14 AMR-associated genes and 76 virulence-associated genes were identified, with little genetic diversity between isolates belonging to the same ST. Several genes including *merR1* which is thought to play a role in ciprofloxacin-resistance were found to be significantly overrepresented in isolates sampled from herds using ampicillin/cloxacillin dry cow therapy. Overall, the presence of resistance genes remains low and current antimicrobial usage patterns do not appear to be driving AMR in *S. aureus* associated with bovine mastitis.

## 1. Introduction

Bovine mastitis remains one of the most economically important diseases affecting the dairy cattle industry worldwide despite intensive research and the increasing uptake of various on-farm control strategies [1,2]. With over 137 different organisms having been associated with mastitis [3], one of the major challenges in controlling bovine mastitis is correctly identifying the pathogen responsible for causing disease to enable appropriate treatment and control options to be developed for a farm. While the relative importance of different pathogens varies amongst farms and countries, more than 90% of intra-mammary infections are caused by only a small number of pathogenic bacteria which are typically classified into two groups; contagious (e.g., *Streptococcus dysgalactiae*, *Streptococcus agalactiae* and *Staphylococcus aureus*) or environmental (e.g., *Escherichia coli* and *Streptococcus uberis*) [4].

In the seasonal calving, pasture-based New Zealand dairy industry, the distribution of mastitis-causing pathogens varies in comparison with many countries in the northern hemisphere, where there is a greater reliance on indoor housing systems and year-round calving practices [5]. Nevertheless, over the last five decades, New Zealand has seen a notable change in the aetiology and epidemiology of mastitis in dairy cows [6,7] with contagious mastitis pathogens, such as *S. agalactiae*, decreasing in prevalence in comparison to environmental pathogens, such as *S. uberis*, which are now more prevalent [8,9]. However, despite the general decline in contagious mastitis-causing pathogens, *S. aureus* continues to cause significant economic losses in the New Zealand dairy industry [6] due largely to its role in sub-clinical and chronic disease resulting in its long-term persistence within many dairy herds [10,11].

Between 20 and 25% of New Zealand *S. aureus* isolates collected from clinical and sub-clinical mastitis cases are resistant to the non-isoxazolyl penicillins (amoxicillin, ampicillin, penicillin) which are commonly used to treat clinical mastitis cases in New Zealand [12,13]. This pattern of resistance raises concerns over the use of antimicrobials for the treatment of mastitis, due to not only the general concern of using antibiotics in food-producing livestock, but also because there is increasing evidence for livestock-associated methicillin-resistant *S. aureus* (LA-MRSA) in human infections [14,15]. These concerns have fuelled a number of studies in New Zealand looking at antimicrobial resistance phenotypes amongst *S. aureus* isolates in cattle [12,13]. To date, resistance to some beta-lactam antimicrobials and trimethoprim/sulfamethoxazole have been found [12], although there are no recent studies that have assessed the presence of MRSA in livestock. Only one LA-MRSA was found amongst 870 bovine *S. aureus* isolates in New Zealand [16], but no widespread surveys have been undertaken, hence the prevalence of LA-MRSA remains undefined. Nevertheless, LA-MRSA has been isolated from several human samples in New Zealand [7], but mainly from individuals who are associated with pig farming or the abattoir industry, or who appear to have acquired MRSA strains from overseas [17]. With current recommendations moving away from the treatment of all cows with antimicrobials at the end of lactation (i.e., ‘blanket dry cow therapy’) towards more targeted dry cow therapy treatments and the use of bismuth subnitrate-based internal teat sealants [18], it is a timely opportunity to review the population structure and antimicrobial susceptibility patterns of mastitis-causing *S. aureus* in New Zealand as well as antimicrobial usage within the dairy industry.

This study aimed to (i) describe the genetic population structure of bovine mastitis-causing *S. aureus* from a small sample of New Zealand dairy herds; providing a snapshot of the genetic basis for both resistance and virulence within the population, and (ii) investigate the association between gene presence-absence and different isolate traits including sample type, antimicrobial susceptibility (AMS), and dry cow therapy (DCT) treatments.

## 2. Materials and Methods

### 2.1. Sample Selection, Microbiology, and Whole-Genome Sequencing

The *S. aureus* isolates used in the present study were all obtained from milk samples collected as part of a previous study for which animal ethics approval had been received and permission was granted by each herd owner for provision of samples for sequencing. The study was not formally reviewed by any of the University’s Human Ethics Committees as the study was judged to be low risk through peer evaluation.

Samples were collected between 22 October 2015 and 27 January 2016 from dairy herds located in the Waikato region of New Zealand’s North Island. Herds were selected on a convenience basis (i.e., the herd owners were willing to be involved in the study), with all invited herd owners having an existing relationship with the research team. Cows in each herd were selected if they presented with either grossly evident changes to the milk and/or heat and swelling of the mammary gland (i.e., clinical mastitis), or an elevated somatic cell count (>200,000 cells/mL) at production recording. The milk samples were collected following aseptic teat end preparation and submitted for routine microbiology following the procedures outlined by the National Mastitis Council [19]. Isolates were defined as *S. aureus* on the basis of being gram-positive cocci, catalase positive and positive on a latex agglutination test (BD BBL Staphyloslide Latex Test Kit, Becton Dickinson, Franklin Lakes, NJ, USA). For this study, a subset of the isolates identified as *S. aureus* were selected for whole-genome sequencing using stratified random sampling within three hierarchical groups to ensure that there was (i) at least one isolate from each herd to explore the between-herd variation in *S. aureus* isolates, (ii) multiple isolates from different cows in the same herd to explore the within-herd variation in *S. aureus* isolates and, (iii) multiple isolates from different quarters on individual cows to explore the within-animal variation in *S. aureus* isolates.

The selected isolates were delivered on Dorset egg slopes (Fort Richard Laboratories, Auckland, New Zealand) to Massey University’s Molecular Epidemiology and Public Health Laboratory (*^m^*EpiLab) where they were re-cultured on Columbia horse blood (CHB) agar plates (Fort Richard Laboratories). From a pure sub-culture, a heavy inoculum was made in nutrient broth No. 2 (Oxoid, Hampshire, UK) containing 15% glycerol and an aliquot frozen at −80 °C. From this, a sub-culture was made on CHB agar and a single bacterial colony was selected for deoxyribonucleic acid (DNA) extraction using a QIAamp DNA Mini Kit (QIAGEN, Hilden, Germany). DNA libraries were prepared using the Nextera XT DNA preparation kit (Illumina, San Diego, CA, USA) for submission to New Zealand Genomics Limited (University of Otago, Dunedin, New Zealand), where 2 × 100 base pair sequencing was performed on the Illumina NextSeq 500 platform following the manufacturer’s instructions.

### 2.2. Genomic Analyses

Raw reads from Illumina sequencing were evaluated, assembled, annotated, and analyzed using the Nullarbor pipeline (*v*2.0; accessed on 27 June 2018) (https://github.com/tseemann/nullarbor). In short, the adapter sequences were trimmed with Trimmomatic (*v*0.38; accessed on 27 June 2018) [20] before undergoing *de novo* assembly in SKESA (*v*2.3; accessed on 27 June 2018) [21], annotation in Prokka (*v*1.13; accessed on 27 June 2018) [22] and scanned using the PubMLST *S. aureus* typing scheme (https://github.com/tseemann/mlst, accessed on 27 June 2018). To generate an alignment of core genome single nucleotide polymorphisms (SNPs), sequence reads for each isolate were aligned to the reference genome MSSA476, a methicillin-susceptible *S. aureus* (MSSA) isolate assigned to sequence type one (ST-1) (GenBank Accession: NC_002953.3) within the Nullarbor pipeline using Snippy (*v*4.0; accessed on 27 June 2018) (https://github.com/tseemann/snippy). Recombinant regions were eliminated from the genome alignment and the remaining polymorphic sites were identified using the software tool Gubbins (*v*2.3.2; accessed on 30 June 2018) [23] before removing indels and extracting SNP sites using the software tool SNP-sites (*v*2.4.0; accessed on 1 July 2018) [24]. Genome Profiler [25] was then used to convert assembly data into whole-genome multilocus sequence typing (wgMLST) allelic profiles and the relationship between isolates was examined using the core SNP alignment to construct a maximum-likelihood phylogeny in R statistical software [26].

In addition to the phylogeny, AMR genes and virulence genes were also identified using the Nullarbor pipeline. For AMR genes the software tool ABRicate (*v*0.8; accessed on 27 June 2018) (https://github.com/tseemann/abricate) was used to screen contigs through both ResFinder (*v*3.1; accessed on 27 June 2018) [27] and the Comprehensive Antibiotic Resistance Database (CARD) [28], whilst screening for virulence genes was undertaken via the Virulence Factors Database [29]. The phylogenetic tree was then displayed alongside the AMR and virulence gene profile of each isolate using the online tool Interactive Tree of Life (iTOL, *v*4.5.3; accessed on 2 July 2018: [30]).

### 2.3. Antimicrobial Susceptibility

The AMS of a subset of the sequenced *S. aureus* isolates described above was determined using a zone diffusion test following the procedures provided by the Clinical and Laboratory Standards Institute (CLSI: [31]). The antimicrobials assessed included penicillin (10 µg), novobiocin (5 µg), cefoxitin (30 μg), tetracycline (30 µg), ceftiofur (30 μg), and oxacillin (1 μg); with isolates being declared ‘susceptible’, ‘intermediate’ or ‘resistant’, based on CLSI recommendations. The susceptibility of each isolate was displayed alongside the phylogenetic tree using iTOL.

### 2.4. Dry Cow Therapy and Antimicrobial Usage

At the time of milk sampling, information was also collected on the use of DCT; that is, the treatment of a cow at the end of lactation with a long-acting antibiotic preparation, with or without a teat sealant. In addition to DCT, the antimicrobial usage (AMU) for each of the study herds was estimated as the daily dose (DD) per cow per lactation from antimicrobial purchase data from the veterinary business servicing the farm for the 2015–2016 lactation. This timeframe was chosen because this is the lactation from which the isolate samples were collected. The DD was calculated for intra-mammary treatments based on tubes per day; for example, if an intra-mammary treatment states two tubes per day on the label and the farm purchased a box of 20 tubes then it was recorded as 10 DDs. The exception to this was dry cow therapy where four tubes (i.e., one tube per quarter at drying off) is considered 1 DD. A similar method was used for injectables but, instead, the recommended volume (in milliliters) per cow per day was considered as the DD and this was divided by the total volume of products sold. For example, a treatment of 20 mL per cow per day would be considered the DD and if 100 mL of a product had been purchased then it was recorded as 5 DDs. An adjustment to the DD was made if the formulation was considered long acting. For example, some oxytetracycline treatments will have three days of activity in a single dose. Thus, a single treatment of 20 mL acting over three days would be equal to a DD of 7 mL. The DDs for all the antimicrobials purchased by a farm over the 12-month period was then summed and divided by the number of cows on-farm to give an average daily dose per cow per lactation.

### 2.5. Statistical Analyses

**Figure 1:**
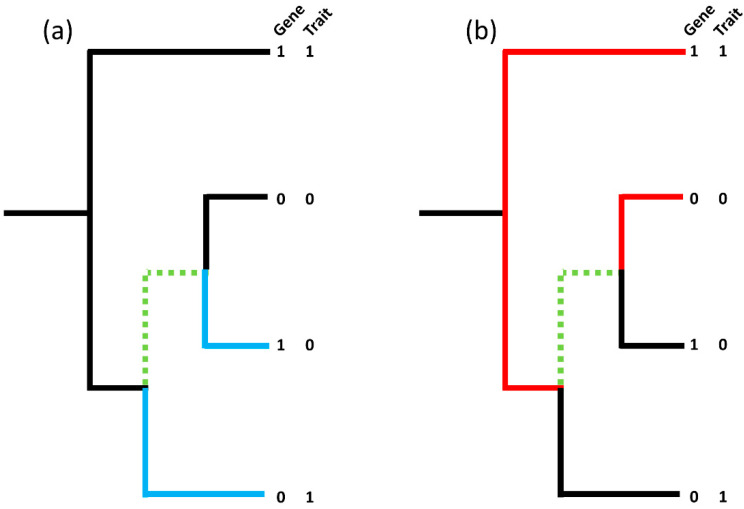
(Adapted from Brynildsrud et al. [32]). (**a**) shows one contrasting pair (blue branches; 1–0|0–1) whilst (**b**) shows another contrasting pair on the same tree (red branches; 1–1|0–0), however, the maximum number of non-intersecting, contrasting pairs in the tree remains as one due to the common branches, highlighted in green, shared by both contrasting pairs. The ‘best’ picking is the red pair with both the gene and the trait present whilst the ‘worst’ picking is the blue pair. To handle confounding by lineage a pairwise comparisons algorithm can be used to identify the maximum number of non-intersecting, contrasting pairs in a tree and calculates the corresponding binomial test *p*-value taking into account the proportion of ‘best’ and ‘worst’ pickings over the set of non-intersecting, contrasting pairs.

The relationship between gene presence-absence and (i) ST, (ii) sample type (i.e., clinical versus subclinical), (iii) AMS, and (iv) DCT treatments, was investigated using the software tool Scoary (*v*1.6.16; accessed on 3 July 2018) [32]. For these analyses, a gene presence-absence matrix was produced in Roary (*v*3.12.0; accessed on 2 July 2018) [33] whilst separate binary matrices indicating the traits of each isolate were produced using Microsoft Excel (2005). For example, in the matrix indicating AMS, ‘1′ would indicate resistance to the antimicrobial whilst ‘0′ would indicate the isolate was susceptible, with any ‘intermediate’ isolates defined as resistant for the purpose of this analysis. A pairwise comparison was then performed in Scoary to find the maximum number of phylogenetically non-intersecting pairs of isolates that contrasted for both the gene and the trait of interest (Figure 1). This type of analysis was performed for each trait to control for any confounding by lineage by accounting for contrasting pairs that share a common ancestor [32,34]. Two criteria for significance were then used to investigate (i) causal inference i.e., genes responsible for membership within a group and, (ii) proportion i.e., genes overrepresented within a group with no causal inference. For casual inferences, genes were found to be significant if the entire range of pairwise comparison *p*-values were less than 0.05. In comparison when looking at proportion, genes were considered significant if they had an odds ratio higher than one, a specificity higher than 95%, and a Benjamini-Hochberg corrected *p*-value [35] below 0.05. For both criteria, any genes annotated as either a ‘hypothetical protein’ or a ‘putative protein’ were removed from subsequent analyses. Significant genes were then used as search terms in the UniProt Knowledgebase (http://uniprot.org/; accessed on 4 July 2018) to determine if the genes were associated with any Gene Ontology terms (http://geneontology.org/; accessed on 4 July 2018) commonly used to describe biological or molecular processes [36]. To investigate the potential relationship between AMU and the presence of resistance genes, a scatterplot was created with AMU plotted against the total number of resistance genes per farm, that is, the sum of all the unique resistance genes identified in each isolate sampled from that farm. A Spearman’s rank correlation coefficient (SCC) could then be calculated using the R package stats [26].

## 3. Results

### 3.1. Genomic Analysis

**Figure 2:**
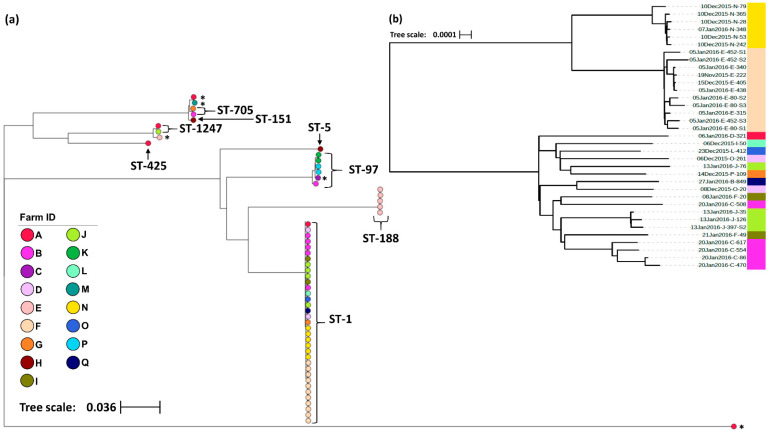
(**a**) Maximum-likelihood phylogeny generated from core single nucleotide polymorphisms across the 57 *S. aureus* isolates. All isolates were collected from bovine raw milk samples in New Zealand and have been labelled with their sequence type (ST) as identified by seven MLST genes. Those isolates with an asterisk (*) indicate isolates whose ST remained undetermined. (**b**) Maximum-likelihood phylogeny generated from core single nucleotide polymorphisms across the 35 *S. aureus* ST-1 isolates identified in (**a**). Isolate IDs identify the date the sample was collected (dd/mm/yyyy), the farm from which it was collected from (A–Q), and the animal ID number. In both trees, the color of the terminal tree nodes also corresponds to the farm from which the isolate was collected. Both figures were created using the online tool Microreact [37].

In total, 57 *S. aureus* isolates were selected for wgMLST analysis with isolates sampled from 51 cows across 17 farms (Table 1). The geographical distribution of farms was limited to the Waikato region of New Zealand’s North Island. In order to protect the identity of the study herds, no map has been presented, however, herds were geographically clustered, with the Euclidean distance between farms ranging from 0.7 Km to 44.1 Km (mean = 19.9 Km). Altogether, 33.3% (19/57) of the isolates were sampled from animals presenting with clinical mastitis compared to 66.7% (38/57) from animals with sub-clinical mastitis. Amongst the 57 isolates, 51,796 polymorphic sites were identified containing 37,746 core SNPs. Eight unique STs were distinguished, with the predominant sequence type ST-1 being found across 64.7% (11/17) of the study farms and 61.5% (32/51) of the sampled animals (Table 1). The maximum-likelihood phylogeny is shown in Figure 2a. However, the relatively large genetic distances between the major clades mask the fine-scale variation between isolates within clades, therefore a higher resolution phylogeny of the ST-1 cluster with 1063 polymorphic sites and 1061 core SNPs identified between the 35 ST-1 isolates has been presented alongside the complete phylogeny (Figure 2b). Overall, four STs, ST-188, ST-5, ST-151 and ST-425, were limited to one farm whilst the remaining, including ST-1, were found across multiple farms. On farms from which multiple isolates were sampled, 50% (6/12) had two or more STs; however, in most cases, the different STs were isolated from different study animals. The exception was one animal with sub-clinical mastitis from which two STs were isolated (ST-1247 and ST-1) from different mammary glands. Across all the remaining isolates, seven STs were identified from sub-clinical samples, whereas four STs were identified from cases of clinical mastitis. The STs from clinical cases were ST-1, ST-97, ST-151 and ST-705 (Table 1).

**Figure 3:**
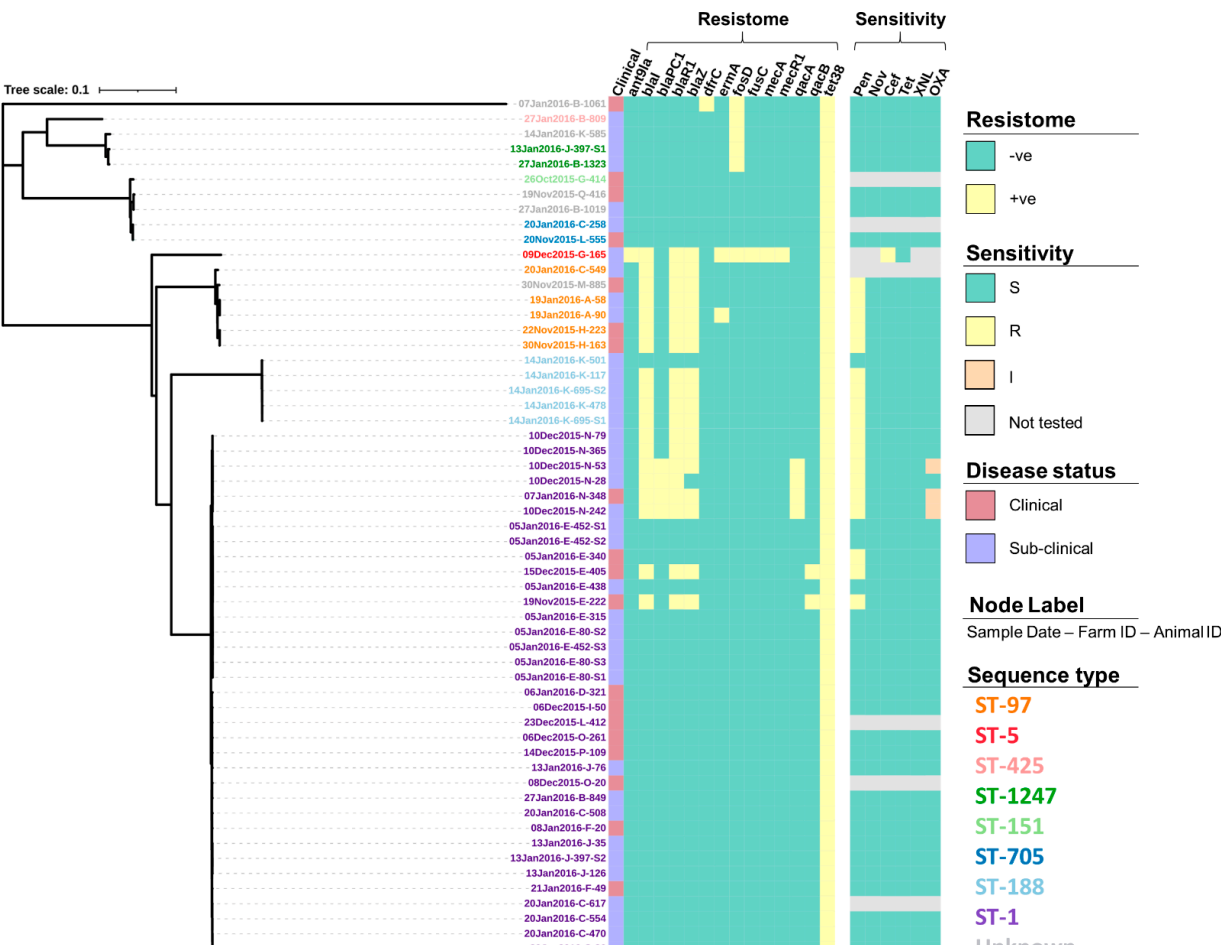
Resistance gene profiles (resistome) and the antimicrobial susceptibility profiles (sensitivity) of 57 *S. aureus* isolates presented alongside a maximum-likelihood tree generated from the core single nucleotide polymorphisms. Susceptibility was determined using a zone diffusion test following the procedures provided by the Clinical and Laboratory Standards Institute (CLSI) for penicillin (Pen), novobiocin (Nov), cefoxitin (Cef), tetracycline (Tet), ceftiofur (XNL), and oxacillin (OXA), with isolates being declared susceptible (S), intermediate (I) or resistant (R), based on CLSI recommendations. Isolate IDs identify the date the sample was collected (dd/mm/yyyy), the farm from which it was collected from (A–Q), and the animal ID number.

**Figure 4:**
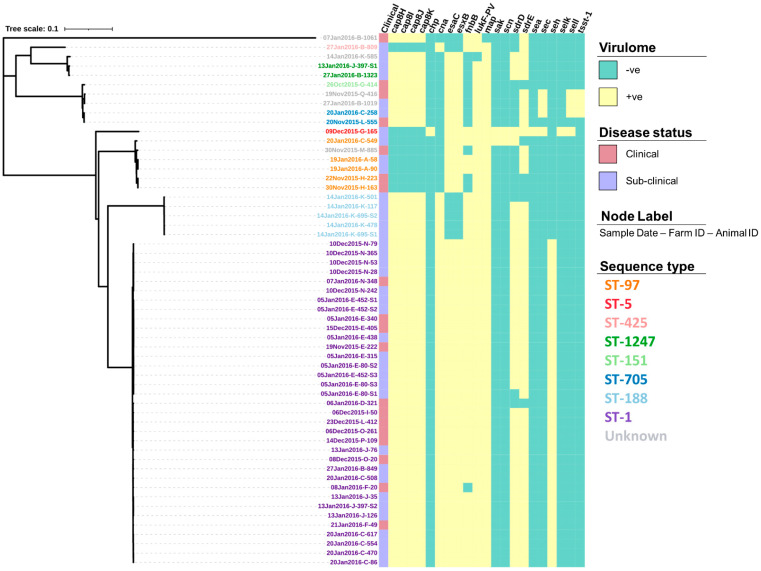
Virulence gene profiles of 57 *S. aureus* isolates presented alongside a maximum-likelihood tree generated from the core single nucleotide polymorphisms. Isolate IDs identify the date the sample was collected (dd/mm/yyyy), the farm from which it was collected from (A–Q), and the animal ID number.

The screening of contigs with ResFinder identified 14 AMR genes (Table S1) associated with a range of drug classes and phenotypes (Table 2). Figure 3 shows how the profile of these genes varied between the isolates. For example, the genes *dfrC*, *fusC*, *mecA* and *mecR1* were only identified in a single isolate. In comparison, the *tet(38)* gene was identified across all 57 isolates. Screening through the Virulence Factors Database identified 76 virulence genes, of which 55 (72.4%) were identified across all 57 isolates (Table S2). The profiles for the 21 remaining virulence genes that varied in prevalence across the isolates can be seen in Figure 4.

### 3.2. Antimicrobial Susceptibility

Overall, 87.7% (50/57) of the *S. aureus* isolates underwent susceptibility testing for all six antimicrobials whilst the single ST-5 isolate was only tested for susceptibility to cefoxitin and tetracycline. Out of those isolates tested against all six antimicrobials, 36.0% (18/50) showed full resistance to penicillin (Figure 3), with the majority of these isolates also shown to have the *blaI* (17/18, 94.4%), *blaR1*(17/18, 94.4%), *blaZ* (16/18, 88.9%), and *tet(38)* (18/18, 100%) genes present as well as a small number also having the *blaPC1* (4/18, 22.2%), *erm(A)* (1/18, 5.6%), *qacA* (4/18, 22.2%), and *qacB* (2/18, 11.1%) genes present (Figure 3). In addition to penicillin, 6.0% (3/50) showed intermediate resistance to oxacillin, with all three isolates (sampled from the same farm) having all the *bla* genes present (*blaI*, *blaR1*, *blaZ*, and *blaPC1*), the *qacA* gene and, the *tet(38)* gene (Figure 3). None of the isolates tested against all six antimicrobials showed any further resistance, however, the single ST-5 isolate that was only tested for susceptibility to cefoxitin and tetracycline did show full resistance to cefoxitin as would be expected from MRSA. This isolate was tested after the initial analysis due to the number of resistance genes that had not been identified in any of the other study isolates including the genes *ant(9)-ia*, *fusC*, *mecA*, and *mecR1*. For the full test results including the zone range for each antimicrobial, readers are directed to Tables S3 and S4.

### 3.3. Dry Cow Therapy and Antimicrobial Usage

Four dry cow therapy treatments were reported across the enrolled herds, namely, Bovaclox^TM^ and Dryclox^®^ (both containing 500 mg cloxacillin and 250 mg ampicillin), Cepravin^®^ (containing 250 mg cephalonium), and Orbenin^®^ (containing 500 mg of cloxacillin). The use of Bovaclox^TM^ was reported by 23.5% (4/17), Cepravin^®^ by 35.3% (6/17), Dryclox^®^ by 17.6% (3/17), and Orbenin^®^ by 47.1% (8/17). In addition to these treatments, the use of Teatseal^®^, a sterile, non-antibiotic intra-mammary infusion was also reported by 58.9% (10/17) of the study herds. Table S5 shows how treatments varied between the isolates that showed some resistance to either penicillin or oxacillin. In the antimicrobial sales data, there was a large amount of heterogeneity in the purchasing of antimicrobials between the study herds with the DD ranging from 0.08 to 3.02 per cow per year (mean = 1.57). However, it is important to note that the DD varied by treatment method, with intra-mammary treatments having the highest DD with a mean of 1.20 followed by injectables with a mean of 0.33, and intra-uterine treatments with a mean of 0.04. Overall, the total number of resistance genes present within a herd was not found to be significantly associated with the herd’s AMU (SCC −0.32, *p* = 0.204, Figure S1).

### 3.4. Statistical Analysis

Across all the Scoary analyses looking at causal inference, no genes were found to be significantly associated with any of the traits of interest, although many candidate genes were identified for each trait. On the other hand, when looking at proportion (see Methods), several significant genes were identified. For example, in the Scoary analysis looking at gene presence-absence and ST, three STs: ST-1, ST-97 and ST-188, were found to be associated with a number of genes. Table 3 highlights the Scoary results for ST-1 which identified 21 genes as being significantly associated with ST. Out of these genes, 85.7% (18/21) were found only in ST-1 isolates. The Gene Ontology terms for these genes revealed that many play a role in DNA replication and modification mechanisms whilst only three, *agrB*, *entH* and *flr*, have been linked to increased virulence or pathogenesis but none to AMR (Table 3). To view all the Scoary analyses looking at gene presence-absence and ST readers are guided to the additional file that is available in the following GitHub repository https://github.com/SSGreening/NZ_S.aureus (accessed on 18 June 2021).

In the Scoary analysis looking at gene presence-absence and sample type, no genes were found to be significantly overrepresented in isolates with either clinical or sub-clinical phenotypes. In the AMS analysis, 12 genes were found overrepresented in isolates showing resistance to penicillin, including the *blaI* and *blaZ* genes identified in ResFinder, as well as a number of other genes thought to play a role in transmembrane activity. In isolates showing intermediate oxacillin resistance, 10 genes were found to be overrepresented with these genes being associated with a range of molecular and biological activities (Table S6). In the analysis looking at gene presence-absence and DCT, no genes were found to be significantly overrepresented in isolates sampled from herds using either Cepravin^®^ or Dryclox^®^. Only one gene, *yezG*, known for its role in the YeeF-YezG toxin-antitoxin module [38], was found to be significantly overrepresented (Benjamini-Hochberg *p*-value = 0.048) in isolates sampled from herds reporting the use of Orbenin^®^. In comparison, 37 genes were found to be overrepresented in isolates sampled from herds reporting the use of Bovaclox^TM^. Out of these genes, 83.8% (31/37) were found across all the isolates sampled from farms using Bovaclox^TM^ including the *merR1* gene, which is thought to be the principal regulatory gene controlling the expression of the *mer* operon responsible for mercury resistance in bacteria [39,40], although it is not known if the rest of the *mer* operon was present or the level of divergence of the *merR1* gene. The *norB4* gene was also found in all the sequenced isolates sampled from farms that had reported using Bovaclox^TM^, and although this gene was not identified by either ResFinder or CARD, it has previously been associated with ciprofloxacin-resistant *S.* aureus [41]. To view the complete Scoary results, readers are guided to the additional file that is available in the following GitHub repository https://github.com/SSGreening/NZ_S.aureus (accessed on 27 June 2018).

## 4. Discussion

Overall, this study identified eight *S. aureus* STs amongst 57 isolates derived from bovine milk samples, with several of them identified from isolates collected both within the same herd and from the same animal. The most prevalent ST found in this study, ST-1, is thought to be derived from ancestral lineages associated with human infection, and previously was uncommon among bovine isolates compared to human MRSA and MSSA isolates [42]. Strikingly the high prevalence of ST-1 in the current study is markedly different from international studies where ST-1 is relatively rare. For example, a recent Swedish study found no ST-1 from amongst 157 bovine *S. aureus* isolates [43], and no ST-1 were found amongst 96 bovine *S. aureus* isolates from 12 European countries [44]. This result is consistent with the growing emergence of human-associated STs as causative agents of bovine mastitis reported in a number of studies worldwide [45,46]. Another study concluded that the majority of bovine *S. aureus* isolates had human ancestral lineage, with the switch from humans to cattle occurring in some cases up to 1500 years ago (e.g., clonal complex 705), or as recently as 23 years ago (clonal complex 5) [44]. This highlights the importance of conducting ongoing genomic surveillance for pathogens such as *S. aureus* to help monitor how it changes over time and understand how these changes may affect the spread and characteristics of the disease. This may involve the analysis of samples taken years prior, similar to this study, in order to build a reference for future analyses For example, if further isolates were to be collected from a greater number of farms, it may be possible to identify if human derived STs are persisting within the New Zealand dairy industry, or whether the sequence types have become bovine adapted.

Further sampling would also help confirm the true prevalence and distribution of different STs as it is likely that the small sample size in this study contributed to the low prevalence of ST-97 and ST-705; the two lineages thought to be otherwise dominant worldwide from isolates derived from bovine milk [42]. The low level of diversity also found between isolates sampled from the same farm may also be due to a strong farm effect whereby transmission bottlenecks associated with predominantly within-farm cow to cow transmission leads to the predominance of particular genotypes derived from a relatively recent common ancestor. This phenomenon further highlights the importance of increasing the number of farms in future studies as well as the geographical spread of farms to ensure the true prevalence and distribution of *S. aureus* has been captured. Herd selection could also be improved to reduce biases. For example, in this study, herds selected were a convenience sample of farmer service by one veterinary clinic. While not selected on the basis of antimicrobial usage or mastitis incidence and hence unlikely to have biased the results of the current study, a truly random sample of herds would increase the external validity of the inferences. It would also be important for future research to sample across species, in particular, any humans in contact with animals or any personnel that may move between farms in order to further explore the relationship between bovine and human *S. aureus* isolates.

Altogether, 14 genes associated with AMR and 76 genes associated with virulence factors were identified with very little variation in the gene profiles both within a single ST and between STs, with the exception of one ST-5 isolate. This ST-5 isolate was the only one found in the study with genes associated with methicillin-resistance namely *mecA* [47] as well as the only isolate that demonstrated resistance to cefoxitin. The infrequency of cefoxitin resistance and absence of genes associated with methicillin resistance fits in with the current understanding that the risk of LA-MRSA originating from cattle is minimal; with only one methicillin-resistant isolate having previously been reported from a bovine source in New Zealand [16]. However, care must be taken when extrapolating the current findings to the wider population of *S. aureus* given that New Zealand lacks a national survey at either the cow or bulk milk level. Only by increasing the number of farms that undergo routine surveillance can there be any confidence that MRSA is not present in the New Zealand dairy industry, especially given the ST-5 isolate in this study that demonstrated resistance to cefoxitin. Out of the remaining 50 (87.7%) isolates that underwent susceptibility testing, very few showed phenotypic evidence for resistance to any of the antimicrobials tested. For example, the *tet(38)* gene, which is associated with resistance to tetracyclines, was found present in all the isolates nevertheless in the zone diffusion tests all isolates were found to be susceptible to tetracycline. These results highlight the limitations in genome-based predictions as gene presence alone doesn’t always confer resistance and the importance of performing phenotypic tests for antimicrobial susceptibility as shown in previous studies [48,49].

Scoary analyses also found several other genes to be overrepresented in those isolates resistant to either penicillin or oxacillin, however, none of these genes, with the exception of the *bla* genes (*blaI*, *blaZ* and, *blaR1*) that are associated with penicillin resistance, are thought to play a role in either pathogen virulence or AMR. Overall, *bla* genes were found present in 33.3% (19/57) of the isolates, the majority of which were identified as ST-5, ST-97 or ST-188. Out of these isolates, 94.7% (18/19) also showed resistance to penicillin. Both this ST profile and susceptibility pattern is consistent with previous studies; for example, in a study by van den Borne and colleagues [50] penicillin resistance was found to be higher in lineages of human origin (including ST-1), although the majority of STs derived from bovine origin such as ST-97, (also identified in the current study), showed little to no resistance in the van den Borne study. A further study by Steele and McDougall [51], in New Zealand, found approximately 45% of *S. aureus* isolates to be both phenotypically penicillin-resistant and genotypically *blaZ* positive, and presence of this genotype/phenotype was associated with very poor bacteriological cure following antimicrobial therapy. This high level of resistance to penicillin in comparison to all the other antimicrobial tested raises some concerns with many penicillins currently regarded as ‘critically important’ (e.g., ampicillin and cloxacillin) by the OIE [52]. This highlights the importance of susceptibility testing in future research to help describe patterns of AMR across New Zealand dairy herds, guide treatment on-farm and, encourage antimicrobial stewardship. Future research should also focus on gathering more detailed descriptions of AMU such as their frequency and reasons for use across a lactation period. Such data would help to provide a more accurate picture of AMU than that based on antimicrobial purchase data alone and would provide a benchmark that could be used to inform priorities and allow measurement of the impact of future stewardship programmes.

In addition to the patterns of AMU and resistance genes, more research into the abundance and distribution of different virulence genes would be useful to compare and contrast *S. aureus* populations across New Zealand. This study showed that there was significant diversity in virulence genes, despite the small sample size. In particular, some of these genes have been associated with food poisoning in humans; namely, a group of highly heat resistant superantigens called staphylococcal enterotoxins (SE) [53]. To date, there are over 20 described SEs with the majority of foodborne illnesses traced to five main serological groups; SEA, SEB, SEC, SED and SEE that are known to survive the pasteurisation process. In this study, the prevalence of SE genes was high, with 70.2% (40/57) of isolates harbouring at least one SE gene whilst 8.8% (5/57) had two or more. This difference is largely due to the association between SEs and STs as all ST-1 isolates, the predominant ST in this study, had the SE gene *seh* present, whilst only ST-705 and ST-5 had multiple SEs present as well as the enterotoxin-like genes *selk* and *sell* and the toxic shock syndrome gene *tsst-1*, all of which have been associated with human disease after the consumption of raw milk [54,55]. However, despite the presence of these genes, it is important that more tests are carried out on samples from the wider population before being able to fully assess the risk to public health; particularly regarding the consumption of raw milk, as previous studies show that their presence alone is not indicative of the level of expression and toxin production in milk [56,57]. By further understanding the patterns of observed virulence genes, it may also help to identify which STs are truly bovine adapted or verses being recent transfers from human populations. For example, the Panton and Valentine Leukotoxin (PVL) virulence genes. *lukS-PV* and *lukF-PV*, are known to be common in many human *S. aureus* strains but absent from bovine strains [58,59]. Similarly, staphylococcal complement inhibitor (*scn*), and chemotaxis inhibitory protein of *S. aureus* (*chp*) are common in human but not bovine isolates [60]. The absence of these human associated virulence factors is also reflected in the study isolates with only 3.5% (2/57) of isolates having the *scn* gene, 1.8% (1/57) having the *chp* gene and none having the *PVL* gene. This suggests that despite there being some historically human associated STs amongst the study isolates, they may be bovine-host adapted.

## 5. Conclusions

By characterising the genetic population structure of mastitis-causing *S. aureus* within a limited number of New Zealand dairy herds, this small study has provided evidence for the predominance of an ST previously associated with human infection. However, despite these STs having historically higher rates of antimicrobial resistance, the presence and diversity of resistance genes remain low. Furthermore, no associations were found between the presence of resistance genes and either AMU or DCT, suggesting that the utility of these treatments has been preserved, except for a small number of penicillin-resistant isolates. This highlights the importance of ongoing research that focuses on describing the genetic population structure of important pathogens such as *S. aureus* as well as the potential use of WGS in guiding clinical decisions around on-farm disease management, especially considering New Zealand’s current goal to eliminate the preventative or metaphylactic use of antimicrobials for in animals by 2030.

## Figures and Tables

**Table 1:** Isolate-level (*n* = 57), farm-level (*n* = 17) and cow-level (*n* = 52) prevalence of eight *Staphylococcus aureus* whole-genome multi-locus sequence types (MLST) isolated from the Waikato region of New Zealand North Island.

Sample Level	Multilocus Sequence Type (ST)							
	ST-1	ST-188	ST-5	ST-705	ST-1247	ST-97	ST-151	ST-425
**Isolates (%)**								
Clinical	12 (63.2)	0	0	1 (5.3)	0	2 (10.5)	1 (5.3)	0
Sub-clinical	23 (60.5)	5 (13.2)	1 (2.6)	1 (2.6)	2 (5.3)	3 (7.9)	0	1 (2.6)
Total	35 (61.4)	5 (8.8)	1 (1.8)	2 (3.5)	2 (3.5)	5 (8.8)	1 (1.8)	1 (1.8)
**Farms (%)**	11 (64.7)	1 (5.9)	1 (5.9)	2 (11.8)	2 (11.8)	3 (17.6)	1 (5.9)	1 (5.9)
**Cows (%)**	32 (61.5)	4 (7.7)	1 (1.9)	2 (3.8)	2 (3.8)	5 (9.6)	1 (1.9)	1 (1.9)

Bold: the bold headings are to clarify at what level prevalence has been calculated as described in the table heading.

**Table 2:** The 14 resistance genes identified in the 57 *S. aureus* isolates with the drug classes influenced by these genes and the drugs common uses in the New Zealand dairy industry. Resistance genes have been grouped by their gene family.

Gene(s)	Drug Class	Common Use in the New Zealand Dairy Industry
*ant(9)-Ia*	Aminoglycosides	Intra-mammary antimicrobials for the treatment of mastitis in lactating cows e.g., neomycin and streptomycin.
*blaI* *blaPC1* *blaR1* *blaZ* *mecA* *mecR1*	ß-lactams	Broad range antimicrobials used to treat a range of intra-mammary, intra-uterine and systemic infections e.g., penicillin, amoxicillin and cloxacillin.
*dfrC*	Diaminopyrimidines	Limited use in cattle, with the exception of Trimethoprim, which is commonly used in combination with sulpha drugs to treat enteric or respiratory tract diseases.
*erm(A)*	Macrolides	Antimicrobials used in the treatment of various systemic and localised bacterial infections including mastitis, respiratory infection, and foot-rot although tilmicosin and tulathromycin have a very long milk withholding period, so are not used in lactating cattle, and rarely on dairy farms whilst erythromycin is also no longer used in cattle in New Zealand.
*fosD*	Fosfomycin	Used to treat a broad variety of bacterial infections in humans, particularly urinary tract infections but it is not registered for animal use in New Zealand.
*fusC*	Fusidic acid	Fusidic acid is not registered for cattle use in New Zealand but has registration for use in dogs.
*qacA* *qacB*	Fluoroquinolones	Injectable antimicrobials used in a range of treatments including *Escherichia coli* and *Pseudomonas* mastitis, osteomyelitis, and respiratory infections, but with very limited usage in the dairy industry.
*tet(38)*	Tetracyclines	Antimicrobial used in the broad-spectrum treatment of local and systemic infections particularly uterine infections and other soft tissue infections in cattle.

**Table 3:** Scoary result summary showing the genes found to be significantly associated with *S. aureus* sequence type (ST)-1 and the Gene Ontology (GO) terms indicating either the biological processes or molecular functions associated with the gene products.

Gene(s)	GO Terms	No. Isolates Gene Present (%)	
		ST-1 (*n* = 35)	Other STs (*n* = 22)
*agrB*	Quorum sensing, pathogenesis, and peptidase activity	35 (100)	0
*entH*	Virulence, metal ion binding, and toxin activity	35 (100)	0
*flr*	Pathogenesis and signal peptide	35 (100)	0
*catE-2*	Transcription regulation and DNA-binding	35 (100)	1 (4.5)
*gltR*		35 (100)	1 (4.5)
*yofA*		35 (100)	0
*gdmA*	Cytolysis and signalling receptor binding	35 (100)	0
*nisC*	Maturation of the lantibiotic	35 (100)	0
*repE/N*	DNA replication initiation and binding	35 (100)	0
*group-2156/7*	Signal peptide	35 (100)	0
*ssbA-1*	DNA replication, repair and recombination, and single-stranded DNA binding	23 (65.7)	0
*ssbA-2*		14 (40.0)	1 (4.5)
*dnaC-2*	DNA replication, helicase activity, synthesis of RNA primers, and ATP binding	21 (60.0)	0
*brnQ-3*	Branched-chain amino acid transmembrane transporter	16 (45.7)	0
*dut*	dUMP biosynthetic process, dUTP activity, and Mg binding	14 (40.0)	0
*bcgIA/B*	DNA modification and N-methyltransferase activity	14 (40.0)	0
*hin*	DNA integration, DNA-binding, and recombinase activity	10 (28.6)	0
*cna*	Pathogenesis, cell adhesion, and collagen binding	9 (25.7)	0

## Data Availability

To view the complete Scoary results, readers are guided to the additional file that is available in the following GitHub repository https://github.com/SSGreening/NZ_S.aureus (accessed on 18 June 2021).

## Supplementary Materials

The following are available online at https://www.mdpi.com/article/10.3390/vetsci8110287/s1. Figure S1: Scatter plot showing the relationship between antimicrobial usage, across 14 dairy herds in the Waikato region of New Zealand’s North Island, and the number of resistant genes found to be present in isolates collected from each farm. The size of each point is proportional to the total number of genes present which the colour indicates which genes were present. AMU: antimicrobial usage, DD: daily dose. Table S1: Prevalence of 14 resistance genes identified across 57 *S. aureus* isolates isolated from bovine raw milk in New Zealand. Genes are listed in ascending order. Table S2: Prevalence of 76 virulence genes identified across 57 *S. aureus* isolates isolated from bovine raw milk in New Zealand. Genes are listed in ascending order with a total of 55 genes being present in 100% of isolates. Table S3: The antimicrobial sensitivity of 51 *S. aureus* isolates derived from bovine milk samples in New Zealand. Sensitivity was determined using a zone diffusion test following the procedures provided by the Clinical and Laboratory Standards Institute. The antimicrobials assessed included penicillin (PEN, 10 μg), novobiocin (NOV, 5 μg), cefoxitin (CEF, 30 μg), tetracycline (TET, 30 μg), ceftiofur (XNL, 30 μg), and oxacillin (OXA, 1 μg); with isolates being declared “sensitive”, “intermediate” or “resistant”, based on CLSI recommendations. NT indicates that the sensitivity to that antimicrobial was not tested. Isolate IDs identify the date the sample was collected (dd/mmm/yyyy), the farm from which it was collected from (A–Q), and the animal ID number (####). Table S4: The presence-absence of 14 resistance genes and the antimicrobial susceptibility across 51 *S. aureus* isolates. Antimicrobial tested zone diffusion includes penicillin (Pen), novobiocin (Nov), cefoxitin (Cef), tetracycline (Tet), ceftiofur (XNL), and oxacillin (OXA); with isolates being declared susceptible (S) or resistant (R) (including those also identified as intermediate), based on CLSI recommendations. Note: one isolate was only tested for Cef and Tet resistance therefore (–) has been used to indicate cases when no isolates tested had the gene present. Table S5: The dry cow therapy treatment reported by 17 farms from which 50 *S. aureus* isolates were sampled and classified as “sensitive”, “intermediate”, or “resistant” to penicillin (PEN, 10 μg) following a zone diffusion test. For the purpose of this analysis, intermediate” isolates have been groups with the “resistant” isolates. Table S6: Scoary result summary showing the genes found to be significantly associated with *S. aureus* isolates showing resistance to either penicillin (*n* = 18) or oxacillin (*n* = 3) and the Gene Ontology (GO) terms indicating either the biological processes or molecular functions associated with the gene products. In total 50 *S. aureus* isolates were included in the analysis with significant genes identified as those with an odds ratio greater than 1, a specificity greater than 95% and, a Benjamini-Hochberg corrected *p*-value below 0.05.
