# Supplementary material for: The Genetic Relatedness and Antimicrobial Resistance Patterns of Mastitis-Causing Staphylococcus aureus Strains Isolated from New Zealand Dairy Cattle
Source: Vet Sci. 2021 Nov 22;8(11):287. doi: 10.3390/vetsci8110287 (PMC8625616; doi:10.3390/vetsci8110287)

Supplementary Materials

The Genetic Relatedness and Antimicrobial Resistance Patterns of Mastitis-Causing *Staphylococcus aureus* Strains Isolated from New Zealand Dairy Cattle

Sabrina S. Greening[1*], Ji Zhang[2,3], Anne C. Midwinter[2], David A. Wilkinson[2,3], Scott McDougall[1,4], M. Carolyn Gates[1], Nigel P. French[2,3]

[1] School of Veterinary Science, Massey University, Palmerston North, New Zealand 4410
[2] Infectious Disease Research Centre, Massey University, Palmerston North, New Zealand 4410
[3] New Zealand Food Safety Science and Research Centre, Hopkirk Research Institute, Massey University, Palmerston North, New Zealand 4410
[4] Cognosco, AnexaFVC, PO Box 21, Morrinsville, New Zealand 3330
* Correspondence: S.Greening@massey.ac.nz

List of tables

Supplementary Table S1. Prevalence of 14 resistance genes identified across 57 *S. aureus* isolated from bovine raw milk in New Zealand. Genes are listed in ascending order 3
isolates

Supplementary Table S2. Prevalence of 76 virulence genes identified across 57 *S. aureus* isolates isolated from bovine raw milk in New Zealand. Genes are listed in ascending order with a total of 55 genes being present in 100% of isolates 4

Supplementary Table S3. The antimicrobial sensitivity of 51 *S. aureus* isolates derived from bovine milk samples in New Zealand. Sensitivity was determined using a zone diffusion test following the procedures provided by the Clinical and Laboratory Standards Institute. The antimicrobials assessed included penicillin (PEN, 10 μg), novobiocin (NOV, 5 μg), cefoxitin (CEF, 30 μg), tetracycline (TET, 30 μg), ceftiofur (XNL, 30 μg), and oxacillin (OXA, 1 μg); with isolates being declared "sensitive", "intermediate" or "resistant", based on CLSI recommendations. NT indicates that the sensitivity to that antimicrobial was not tested. Isolate IDs identify the date the sample was collected (dd/mmm/yyyy), the farm from which it was collected from (A-Q), and the animal ID number (####). 5

Supplementray Table S4. The presence-absence of 14 resistance genes and the antimicrobial susceptibility across 51 *S. aureus* isolates. Antimicrobial tested zone diffusion includes penicillin (Pen), novobiocin (Nov), cefoxitin (Cef), tetracycline (Tet), ceftiofur (XNL), and oxacillin (OXA); with isolates being declared susceptible (S) or resistant (R) (including those also identified as

intermediate), based on CLSI recommendations. Note: one isolate was only tested for *Cef* and *Tet* resistance therefore (–) has been used to indicate cases when no isolates tested had the gene present..7

Supplementary Table S5. The dry cow therapy treatment reported by 17 farms from which 50 S. aureus isolates were sampled and classified as “sensitive”, “intermediate”, or “resistant” to penicillin (PEN, 10 μg) following a zone diffusion test. For the purpose of this analysis, intermediate” isolates have been groups with the “resistant” isolates. 8

Supplementary Table S6. Scoary result summary showing the genes found to be significantly associated with *S. aureus* isolates showing resistance to either penicillin (n=18) or oxacillin (n=3) and the Gene Ontology (GO) terms indicating either the biological processes or molecular functions associated with the gene products. In total 50 *S. aureus* isolates were included in the analysis with significant genes identified as those with an odds ratio greater than 1, a specificity greater than 95% and, a Benjamini-Hochberg corrected p-value below 0.05.. 9

List of Figures

Supplementary Figure S1. Scatter plot showing the relationship between antimicrobial usage, across 14 dairy herds in the Waikato region of New Zealand’s North Island, and the number of resistant genes found to be present in isolates collected from each farm. The size of each point is proportional to the total number of genes present which the colour indicates which genes were present. AMU: antimicrobial usage, DD: daily dose...11

Supplementary Table S1. Prevalence of 14 resistance genes identified across 57 *S. aureus* isolates isolated from bovine raw milk in New Zealand. Genes are listed in ascending order

**Resistance Gene(s)**	**Number of Isolate (%)**
ant(9)-Ia, dfrC, fusC, mecA, mecR1	1 (1.75)
erm(A), qacB	2 (3.51)
blaPC1, qacA	4 (7.02)
fosD	6 (10.53)
blaZ	18 (31.58)
blaI, blaR1	19 (33.33)
tet(38)	57 (100.0)

Supplementary Table S2. Prevalence of 76 virulence genes identified across 57 *S. aureus* isolates isolated from bovine raw milk in New Zealand. Genes are listed in ascending order with a total of 55 genes being present in 100% of isolates

**Virulence Gene(s)**	**Number of Isolates (%)**
chp, sea, selk	1 (1.75)
sak, scn	2 (3.51)
tsst-1	3 (5.26)
sec, sell	4 (7.02)
She	35 (61.40)
Can	41 (71.93)
SdrD	42 (73.68)
FnbB	45 (78.95)
cap8(H-K)	49 (85.96)
esaC, esxB	50 (87.72)
SdrE	51 (89.47)
lukF-PV, map	56 (98.25)
adsA, aur, cap8(A-G), cap8(L-P), clf(A,B,P) coa, ebp, esa(A-B), (essA-C) esxA, fnbA, geh, hlb, hld, hlg(A-C), hly/hla, hysA, ica(A-D,R), isd(A-G), lip, sbi, sdrC, spa, srtB, ssp(A-C), vWbp	57 (100.0)

Supplementary Table S3. The antimicrobial sensitivity of 51 *S. aureus* isolates derived from bovine milk samples in New Zealand. Sensitivity was determined using a zone diffusion test following the procedures provided by the Clinical and Laboratory Standards Institute. The antimicrobials assessed included penicillin (PEN, 10 µg), novobiocin (NOV, 5 µg), cefoxitin (CEF, 30 µg), tetracycline (TET, 30 µg), ceftiofur (XNL, 30 µg), and oxacillin (OXA, 1 µg); with isolates being declared “sensitive”, “intermediate” or “resistant”, based on CLSI recommendations**.** NT indicates that the sensitivity to that antimicrobial was not tested. Isolate IDs identify the date the sample was collected (dd/mmm/yyyy), the farm from which it was collected from (A-Q), and the animal ID number (####).

**Isolate ID**	**Sample type**	**Diameter (mm) (Sensitivity)**					
		**PEN**	**NOV**	**CEF**	**TET**	**XNL**	**OXA**
19Jan2016-A-90	Subclinical	18 (R)	36 (S)	30 (S)	31 (S)	32 (S)	21 (S)
27Jan2016-B-849	Subclinical	44 (S)	32 (S)	28 (S)	30 (S)	30 (S)	22 (S)
27Jan2016-B-1019	Subclinical	45 (S)	32 (S)	28 (S)	29 (S)	33 (S)	28 (S)
20Jan2016-C-554	Subclinical	46 (S)	40 (S)	28 (S)	32 (S)	34 (S)	26 (S)
07Jan2016-B-1061	Clinical	46 (S)	35 (S)	28 (S)	26 (S)	32 (S)	26 (S)
06Jan2016-D-321	Clinical	40 (S)	40 (S)	30 (S)	32 (S)	30 (S)	24 (S)
05Jan2016-E-452	Subclinical	44 (S)	36 (S)	29 (S)	30 (S)	32 (S)	26 (S)
27Jan2016-B-1323	Subclinical	40 (S)	32 (S)	28 (S)	32 (S)	32 (S)	22 (S)
20Jan2016-C-86	Subclinical	40 (S)	34 (S)	28 (S)	26 (S)	30 (S)	24 (S)
05Jan2016-E-452	Subclinical	44 (S)	36 (S)	28 (S)	30 (S)	32 (S)	25 (S)
08Jan2016-F-20	Clinical	40 (S)	36 (S)	26 (S)	29 (S)	36 (S)	24 (S)
21Jan2016-F-49	Clinical	42 (S)	38 (S)	28 (S)	31 (S)	31 (S)	24 (S)
05Jan2016-E-452	Subclinical	50 (S)	38 (S)	29 (S)	26 (S)	32 (S)	28 (S)
30Nov2015-H-163	Clinical	26 (R)	35 (S)	28 (S)	34 (S)	35 (S)	23 (S)
06Dec2015-I-50	Clinical	38 (S)	35 (S)	28 (S)	28 (S)	35 (S)	26 (S)
09Dec2015-G-165	Subclinical	NT	NT	10 (R)	(S)	NT	NT
05Jan2016-E-80	Subclinical	40 (S)	34 (S)	27 (S)	26 (S)	30 (S)	26 (S)
13Jan2016-J-35	Subclinical	38 (S)	36 (S)	27 (S)	30 (S)	32 (S)	24 (S)
05Jan2016-E-80	Subclinical	44 (S)	36 (S)	26 (S)	27 (S)	30 (S)	26 (S)
13Jan2016-J-76	Subclinical	38 (S)	35 (S)	28 (S)	31 (S)	31 (S)	24 (S)
05Jan2016-E-80	Subclinical	42 (S)	36 (S)	26 (S)	28 (S)	31 (S)	26 (S)
13Jan2016-J-126	Subclinical	40 (S)	36 (S)	28 (S)	29 (S)	32 (S)	24 (S)
19Nov2015-E-222	Clinical	21 (R)	36 (S)	29 (S)	31 (S)	36 (S)	24 (S)
14Jan2016-K-695	Subclinical	11 (R)	34 (S)	28 (S)	29 (S)	27 (S)	16 (S)
13Jan2016-J-397	Subclinical	40 (S)	35 (S)	26 (S)	29 (S)	30 (S)	24 (S)
14Jan2016-K-501	Subclinical	40 (S)	35 (S)	30 (S)	29 (S)	29 (S)	26 (S)
13Jan2016-J-397	Subclinical	46 (S)	37 (S)	27 (S)	30 (S)	30 (S)	24 (S)
05Jan2016-E-315	Subclinical	41 (S)	34 (S)	27 (S)	30 (S)	30 (S)	26 (S)
15Dec2015-E-405	Clinical	22 (R)	34 (S)	28 (S)	26 (S)	30 (S)	25 (S)

Table S3 continues next page.

Table S3 continued.

Isolate ID	Sample type	Diameter (mm) (Sensitivity)					
		**PEN**	**NOV**	**CEF**	**TET**	**XNL**	**OXA**
05Jan2016-E-438	Subclinical	44 (S)	36 (S)	28 (S)	28 (S)	30 (S)	25 (S)
05Jan2016-E-340	Clinical	22 (R)	34 (S)	26 (S)	28 (S)	30 (S)	24 (S)
20Nov2015-L-555	Clinical	49 (S)	36 (S)	28 (S)	30 (S)	33 (S)	30 (S)
14Jan2016-K-585	Subclinical	44 (S)	37 (S)	27 (S)	32 (S)	34 (S)	30 (S)
30Nov2015-M-885	Clinical	21 (R)	37 (S)	28 (S)	30 (S)	37 (S)	22 (S)
10Dec2015-N-28	Subclinical	16 (R)	36 (S)	26 (S)	26 (S)	36 (S)	19 (S)
14Jan2016-K-695	Subclinical	11 (R)	34 (S)	28 (S)	29 (S)	27 (S)	16 (S)
10Dec2015-N-53	Subclinical	14 (R)	36 (S)	28 (S)	30 (S)	26 (S)	12 (I)
22Nov2015-H-223	Clinical	19 (R)	40 (S)	30 (S)	33 (S)	40 (S)	22 (S)
10Dec2015-N-79	Subclinical	18 (R)	38 (S)	29 (S)	30 (S)	32 (S)	20 (S)
06Dec2015-O-261	Clinical	40 (S)	34 (S)	26 (S)	30 (S)	34 (S)	28 (S)
10Dec2015-N-242	Subclinical	12 (R)	30 (S)	27 (S)	28 (S)	25 (S)	12 (I)
07Jan2016-N-348	Clinical	14 (R)	26 (S)	28 (S)	30 (S)	25 (S)	12 (I)
10Dec2015-N-365	Subclinical	15 (R)	31 (S)	27 (S)	30 (S)	30 (S)	19 (S)
14Jan2016-K-117	Subclinical	14 (R)	34 (S)	29 (S)	27 (S)	29 (S)	15 (S)
14Dec2015-P-109	Clinical	34 (S)	36 (S)	28 (S)	30 (S)	30 (S)	28 (S)
20Jan2016-C-470	Subclinical	44 (S)	34 (S)	26 (S)	28 (S)	30 (S)	26 (S)
14Jan2016-K-478	Subclinical	13 (R)	34 (S)	29 (S)	28 (S)	29 (S)	16 (S)
19Nov2015-Q-416	Clinical	50 (S)	36 (S)	28 (S)	30 (S)	36 (S)	31 (S)
20Jan2016-C-508	Subclinical	50 (S)	36 (S)	34 (S)	32 (S)	34 (S)	30 (S)
19Jan2016-A-58	Subclinical	19 (R)	35 (S)	28 (S)	32 (S)	31 (S)	22 (S)
27Jan2016-B-809	Subclinical	44 (S)	34 (S)	27 (S)	28 (S)	30 (S)	26 (S)

Supplementray Table S4. The presence-absence of 14 resistance genes and the antimicrobial susceptibility across 51 *S. aureus* isolates. Antimicrobial tested zone diffusion includes penicillin (Pen), novobiocin (Nov), cefoxitin (Cef), tetracycline (Tet), ceftiofur (XNL), and oxacillin (OXA); with isolates being declared susceptible (S) or resistant (R) (including those also identified as intermediate), based on CLSI recommendations. Note: one isolate was only tested for *Cef* and *Tet* resistance therefore (–) has been used to indicate cases when no isolates tested had the gene present.

**Gene presence/absence**		**Pen**		**Nov**		**Cef**		**Tet**		**XNL**		**OXA**	
**(no. isolates with gene)**		**R**	**S**	**R**	**S**	**R**	**S**	**R**	**S**	**R**	**S**	**R**	**S**
ant(9)-ia	*Absent*	18	32	0	50	0	50	0	50	0	50	3	47
**(n = 1)**	*Present*	-	-	-	-	1	0	0	1	-	-	-	-
blaI	*Absent*	1	32	0	33	0	33	0	33	0	33	0	33
**(n = 18)**	*Present*	17	0	0	17	1	17	0	18	0	17	3	14
blaPC1	*Absent*	14	32	0	46	0	46	0	46	0	46	0	46
**(n = 4)**	*Present*	4	0	0	4	0	4	0	4	0	4	3	1
blaR1	*Absent*	1	32	0	33	0	33	0	33	0	33	0	33
**(n = 18)**	*Present*	17	0	0	17	1	17	0	18	0	17	3	14
blaZ	*Absent*	2	32	0	34	0	34	0	34	0	46	0	34
**(n = 17)**	*Present*	16	0	0	16	1	16	0	17	0	4	3	13
dfrC	*Absent*	18	31	0	49	0	49	0	49	0	33	0	34
**(n = 1)**	*Present*	0	1	0	1	0	1	0	1	0	17	3	13
erm(A)	*Absent*	17	32	0	49	0	49	0	49	0	34	3	46
**(n = 2)**	*Present*	1	0	0	1	1	1	0	2	0	16	0	1
fosD	*Absent*	18	27	0	45	0	45	0	45	0	49	3	42
**(n = 6)**	*Present*	0	5	0	5	1	5	0	6	0	1	0	5
fusC	*Absent*	18	32	0	50	0	50	0	50	0	50	3	47
**(n = 1)**	*Present*	-	-	-	-	1	0	0	1	-	-	-	-
mecA	*Absent*	18	32	0	50	0	50	0	50	0	50	3	47
**(n = 1)**	*Present*	-	-	-	-	1	0	0	1	-	-	-	-
mecR1	*Absent*	18	32	0	50	0	50	0	50	0	50	3	47
**(n = 1)**	*Present*	-	-	-	-	1	0	0	1	-	-	-	-
qacA	*Absent*	14	32	0	46	0	46	0	46	0	46	0	46
**(n = 4)**	*Present*	4	0	0	4	0	4	0	4	0	4	3	1
qacB	*Absent*	16	32	0	48	0	48	0	48	0	48	3	45
**(n = 2)**	*Present*	2	0	0	2	0	2	0	2	0	2	0	2
tet(38)	*Absent*	0	0	0	0	0	0	0	0	0	0	0	0
**(n = 51)**	*Present*	18	32	0	50	0	50	0	50	0	50	3	47

Supplementary Table S5. The dry cow therapy treatment reported by 17 farms from which 50 *S. aureus* isolates were sampled and classified as "sensitive", "intermediate", or "resistant" to penicillin (PEN, 10 µg) following a zone diffusion test. For the purpose of this analysis, intermediate" isolates have been groups with the "resistant" isolates.

			**Number of animals (% treated)**			
**Classification**	***Antimicrobial***	***n***	**Bovaclox**	**Dryclox**	**Cepravin**	**Orbenin**
**Resistant**	PEN	18	4 (22.2)	0 (0.00)	12 (66.7)	4 (22.2)
	OXA	3	0 (0.00)	0 (0.00)	3 (100)	0 (0.00)
**Sensitive**	PEN	32	1 (3.1)	5 (15.6)	0 (0.00)	20 (62.5)
	OXA	47	8 (17.0)	6 (12.8)	18 (38.3)	24 (51.1)

Supplementary Table S6. Scoary result summary showing the genes found to be significantly associated with *S. aureus* isolates showing resistance to either penicillin (n=18) or oxacillin (n=3) and the Gene Ontology (GO) terms indicating either the biological processes or molecular functions associated with the gene products. In total 50 *S. aureus* isolates were included in the analysis with significant genes identified as those with an odds ratio greater than 1, a specificity greater than 95% and, a Benjamini-Hochberg corrected p-value below 0.05.

**Gene**	**GO terms**	**Number of isolates gene present (%)**	
		**PEN resistant**	**PEN sensitive**
blaI	DNA binding, gene expression and, penicillinase repressor	16 (88.9)	0
blaZ	Betalactamase and hydrolase activity	10 (55.6)	0
xerC-2	DNA recombination, integration and	10 (55.6)	0
xerC-5	binding and, cell division	10 (55.6)	0
setA	Sugar efflux transporter	9 (50.0)	1 (3.1)
group-1603	DNA binding and isomerase activity	7 (38.9)	0
blaR1	Regulatory protein blaR1, penicillin binding and, cell membrane component	7 (38.9)	0
dpiB	Cell membrane component, protein histidine kinase activity and, transferase activity	8 (44.4)	1 (3.1)
group-1117	Transmembrane transport activity	6 (33.3)	0
group-1840	Cell membrane component and hydrolase activity	6 (33.3)	0
group-3090	Transmembrane transport activity and NADH dehydrogenase	6 (33.3)	0
qacR	DNA binding and transcriptional regulator	6 (33.3)	0

Table S6 continues next page.

Table S6 continued.

**Gene**	**GO terms**	**Number of isolates gene present (%)**	
		**OXA resistant**	**OXA sensitive**
group-1669	Peptidase and hydrolase activity, proteolysis and, pathogenesis	3 (100)	1 (2.1)
dauA	Transmembrane transport activity	3 (100)	1 (2.1)
nhaX-1	Transcription regulation	3 (100)	1 (2.1)
group-3291	Regulatory protein BlaR1	3 (100)	1 (2.1)
group-112	Transferase and kinase activity, metal ion binding and, phosphorylation	3 (100)	2 (4.3)
clpE	ATP binding and peptidase activity	3 (100)	2 (4.3)
group-3310	Isomerase and catalytic activity and, gluconeogenesis	3 (100)	2 (4.3)
tcaA-2	Cell membrane component	3 (100)	2 (4.3)
hicB	Antitoxin	3 (100)	2 (4.3)
dinB-2	DNA binding, replication and repair, transferase activity and, metal ion binding	3 (100)	2 (4.3)

Supplementary Figure S1. Scatter plot showing the relationship between antimicrobial usage, across 14 dairy herds in the Waikato region of New Zealand's North Island, and the number of resistant genes found to be present in isolates collected from each farm. The size of each point is proportional to the total number of genes present which the colour indicates which genes were present. AMU: antimicrobial usage, DD: daily dose.

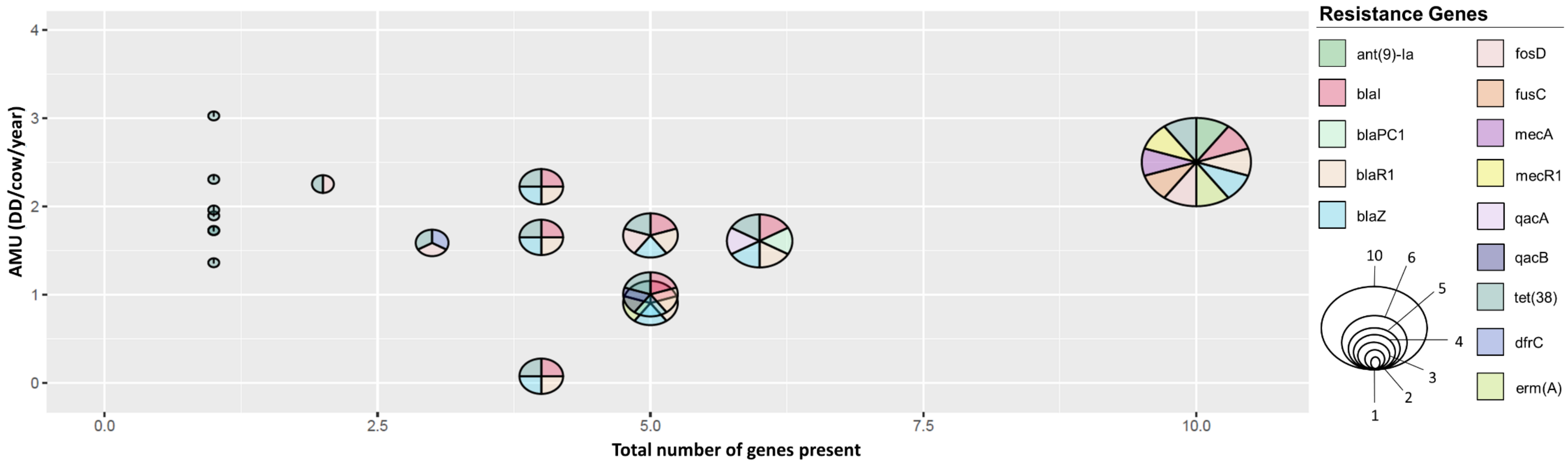

Supplement: Supplementary file 1 [file vetsci-08-00287-s001.zip › vetsci-1441157-supplementary.pdf]
